# Goal-Side Selection in Soccer Penalty Kicking When Viewing Natural Scenes

**DOI:** 10.3389/fpsyg.2012.00312

**Published:** 2012-09-06

**Authors:** Matthias Weigelt, Daniel Memmert

**Affiliations:** ^1^Department of Sport and Health, University of PaderbornPaderborn, Germany; ^2^German Sports University Cologne, Institute of Cognitive and Team/Racket Sport ResearchCologne, Germany

**Keywords:** anticipation, implicit action priming, action selection, sport performance, soccer penalty

## Abstract

The present study investigates the influence of goalkeeper displacement on goal-side selection in soccer penalty kicking. Facing a penalty situation, participants viewed photo-realistic images of a goalkeeper and a soccer goal. In the action selection task, they were asked to kick to the greater goal-side, and in the perception task, they indicated the position of the goalkeeper on the goal line. To this end, the goalkeeper was depicted in a regular goalkeeping posture, standing either in the exact middle of the goal or being displaced at different distances to the left or right of the goal’s center. Results showed that the goalkeeper’s position on the goal line systematically affected goal-side selection, even when participants were not aware of the displacement. These findings provide further support for the notion that the implicit processing of the stimulus layout in natural scenes can effect action selection in complex environments, such in soccer penalty shooting.

## Introduction

The ability to anticipate the consequences of ones’ own actions and of the actions of other co-actors is an essential part of social interaction. Such anticipation skills are especially important for decision making in complex environments, such as in a sport setting like the soccer penalty kick, which has become one of the most prominent paradigms to investigate anticipation skills in sports (e.g., McGarry and Franks, 2000; Savelsbergh et al., 2005; Dicks et al., 2010a). In penalty kicking, two strategies have been observed for kickers when it comes to the selection of the left or right goal-side (e.g., Van der Kamp, 2006). The kicker can either anticipate the goal corner in advance and thus, select the goal-side independently of the goalkeeper, or base the decision on observing the goalkeeper’s action (i.e., his/her jump direction) and react late during the execution of the penalty. An anticipation strategy in which the kicker selects the goal-side in advance has proven to be more successful than reacting to the goalkeeper’s action during the run-up (Van der Kamp, 2006). From this finding, the question central to the present study arises: What kind of information do penalty kickers use to select their actions (i.e., the left or right goal-side) in advance? As we will show, these complex decisions are based on the visual processing of the action environment and basic spatial judgment.

Before taking a closer look at the visual information processes upon which the soccer kicker selects his/her goal corner during the penalty kick, we first consider the prominent theory of anticipatory behavioral control (ABC), advanced by Hoffmann (1993, 2009). According to this theory, peoples’ anticipations are based on the acquisition of action-effect (A-E) representations. These A-E representations become stronger the more often a certain action leads to the desired effect. In soccer, the player learns the contingency between kicking a soccer ball in a certain way (e.g., instep kick) and the direction and/or trajectory of the ball’s flight after the impact. Within the ABC-theory this is thought of “as being the primary learning process in the acquisition of behavioral competence” (Hoffmann, 2009, p. 22). Once the A-E representation is established, anticipating a certain effect (e.g., scoring on the left goal-side) will activate the appropriate action (e.g., instep kick with a small body rotation to the left while kicking with the inside of the right foot).

However, voluntary actions are not only linked to effect representations (Hommel et al., 2001), but also to the situational context in which a desired effect is consistently realized by a certain action. In fact, specific situational features become integrated into existing A-E representations. Hoffmann (2009, p. 22) considers this conditionalization of A-E representations as “being a secondary learning process.” Importantly, conditionalized A-E representations will be *directly triggered*, when the situational features correspond to the represented condition (i.e., the situational context). This notion can be traced back to Lewin (1928), who used the German term “Aufforderungscharakter,” to Ach (1913), who spoke of “voluntive Objektion,” and/or to Gibson (1979), who proposed that objects in the environment provide “affordances” to act in a particular way. Together, these conceptions suggest that situational features trigger a certain habitual behavior, as long as people act in a specific context (e.g., soccer penalty).

Applied to the penalty situation in soccer, successful performance does not only rely on excellent kicking skills, which are based on well-established A-E representations, but also on sufficient information uptake during the visual processing of the environment, which is based on the continuous integration of situational features into existing A-E representations. During the penalty kick, processing a specific situational feature, such as the position of the goalkeeper on the goal line, may activate the corresponding A-E representation and thus, trigger a certain action in the kicker. For example, if the goalkeeper stands more on the right side of the goal, leaving a larger area on the left side uncovered, the kicker will kick to the left goal-side. Hence, when examining people’s decision making in complex environments, one has to also consider the situational context in which the actions are carried out. This means that in the soccer penalty situation, anticipating the outcome of the kick (e.g., scoring on the left or right goal-side) does not only depend on good anticipation skills, but also on the sufficient processing of situational features (e.g., the exact goalkeeper’s position on the goal line), which may trigger the corresponding A-E representations (e.g., a certain kicking behavior). The more general aim of the present study is therefore to investigate if goal-side selection (drawing on A-E representations) in soccer penalty kicking is influenced by the goalkeepers’ position on the goal line (drawing on the processing of situational features).

The present study has been largely motivated by a recent study of Masters et al. (2007). In a video analysis of 200 penalty kicks in high-level soccer competitions (e.g., World Cups, European Championships, Africa Cup of Nations etc.), these authors observed that goalkeepers do not stand in the exact middle of the goal line in 96% of kicks, while, at the same time, professional kickers reliably select the greater goal-side. This observation led them to the question whether the penalty takers consciously perceived the goalkeeper’s displacement or whether the selection of the greater goal-side was the result of implicit priming. To answer this question, Masters et al. (2007) designed three ingenious laboratory experiments with the aim to replicate and isolate the effects under controlled conditions. In Experiment 1, participants viewed a filled block, which was presented at different displacements to the left or right of a rectangle’s center (scaled to 3% of a regular soccer goal). Participant’s task was to indicate the larger area to the side of the rectangle. In Experiment 2, the filled block was replaced by an image of Oliver Kahn and the set-up was scaled to 44% of a regular soccer goal. The task for the participants was now to kick a soccer ball to the greater side of the goal (i.e., the side with the greater area). The same set-up was used in Experiment 3, but this time, participants were asked to only kick to the goal when they perceived the goalkeeper to be standing in the middle of the goal. The results demonstrated that participants were able to reliably indicate the greater area of the rectangle (Experiment 1) and to direct their kicks to the greater side of the goal (Experiment 2), even when executing the kick meant to indicate that they perceived the goalkeeper to be standing in goal center (Experiment 3).

Masters et al. (2007) related their findings to the empirical law of sensation from psychophysics, also known as the Weber–Fechner law (cf. Krueger, 1989). Essentially, this law captures the relationship between the (objective) physical world and the (subjective) psychological world of perception, and describes the just-noticeable difference between two physical stimuli varied along a single dimension (e.g., visual, auditory, tactile etc.). Following the empirical law of sensation, the difference of two physical stimuli can only be perceived when it overcomes a differential threshold. Applied to the penalty situation, whether or not the kicker will recognize the shift of the goalkeeper depends on the size relation between goal and goalkeeper, as well as on the viewing distance of the penalty kicker. Participants in Masters et al.’s study directed their kicks more often to the greater goal-side already for goalkeeper displacements of only 0.5%. This effect of implicit priming on goal-side selection was independent of the size of the stimulus display (scaled to 3 vs. 44% of the real penalty situation) and corresponded to the just-noticeable difference reliably found in line-bisection studies (e.g., Jewell and McCourt, 2000). The most surprising aspect of the findings was, however, that participants only became aware of the displacement when the goalkeeper position was shifted by 3% on the goal line. Hence, goal-side selection was driven by perceptual discriminations that were not consciously perceived by the participants.

On the basis of the original study by Masters et al. (2007), the present experiment investigates the effect of implicit priming on goal-side selection when presenting a natural scene (i.e., a goalkeeper in real soccer goal) to the kicker. In the original study, degraded stimulus images were used that consisted of a rectangle, which represented the goal, and a filled block (Experiment 1) or an image of Oliver Kahn (Experiments 2 and 3), which represented the goalkeeper. Interestingly, when looking at the image of Oliver Kahn, it appears that he was either shown with his arms behind the back or without arm. In any event, these stimuli did not represent the natural environment of the penalty situation, because in a regular game, the penalty kicker does not simply kick to a rectangle and at the same time, the goalkeeper does not passively await the kick with her/his arms behind her/his back. It is therefore the question whether a similar pattern of goal-side selection can be found when using photo-realistic images of the penalty situation.

This extension of the stimulus material to photo-realistic stimuli is in line with current approaches to investigate the mechanisms of the visual system in natural scenes (for a review see Felsen and Dan, 2005). Most of our knowledge about the visual system is gathered from experiments using simple stimuli, either displaying bright spots on dark backgrounds, or dark spots on light background. This methodological approach of simplistic stimulus presentation has also been used in line-bisection tasks (e.g., Lindell et al., 2007), and in the soccer penalty study by Masters et al. (2007) described above. Line-bisection tasks have been mostly used in basic research on visuospatial neglect (Lindell et al., 2007) and pseudoneglect (Jewell and McCourt, 2000). Recently, line-bisection performance has been directly related to more complex natural environments (Nicholls et al., 2010). These natural environments, in which people carry out our actions, however, are made up of rich colors and a distinct spatial structure. Importantly, the visual system has adapted to process the characteristic visual properties of natural scenes (Simoncelli and Olshausen, 2001; Kayser et al., 2004). In fact, there is evidence that the stimulus types often used in laboratory experiments are not representative of natural viewing behavior (Dorr et al., 2010). For example, when people look at natural scenes, their saccadic latencies are significantly shorter, allowing for faster reactions to potentially critical stimuli (White et al., 2008). Also, color information facilitates the processing of natural scenes (Delorme et al., 1999). Thus, from a methodological point of view, it is important to examine whether the results obtained from simple stimulus presentations [i.e., filled block (Experiment 1) and degraded black and white image of Oliver Kahn (Experiments 2 and 3) depicted on a white rectangle] by Masters et al. (2007) extend to task-contexts in which photo-realistic images of natural stimuli (i.e., real-world penalty scenario) are used.

To this end, photo-realistic stimulus images of a regular soccer goal and a goalkeeper standing on the goal line in a neutral goalkeeping posture were used in the present study. Specifically, the goalkeeper was displayed in a parallel stance with his knees slightly bend, his arms in a “ready-to-catch” position on the side, and the gaze straight ahead, focusing on the penalty taker. The goalkeeper was positioned on the goal line at different displacements and participant’s task was to kick to the side with the greater goal area in the first part of the experiment. A stimulus image with the goalkeeper standing in the exact middle on the goal line was also included into the experiment (a condition not present in the original study by Masters et al., 2007). This was done to examine a potential bias of kicking to either the left or right side of the goal.

One general and one specific prediction were made for the present experiment. The general prediction referred to the goalkeeper’s displacement and stated that participants would direct more kicks to the greater goal-side, even under conditions in which they were not aware of the displacement. If true, this would show that a similar pattern of goal-side selection can be found when using photo-realistic images of the penalty situation. The specific prediction related to the inclusion of the condition in which the goalkeeper was presented in the exact middle of the goal. Here, it is predicted that the right-footed participants of the present study select the right goal-side more often than the left goal-side. This prediction is based (purely) on inferences from the biomechanics of kicking. Accordingly, right-footed players will approach the ball from the left side, resulting in a run-up direction to the right. Continuing to kick the ball to the right goal-side is then easier than changing the kicking direction to the left goal-side. Surprisingly, and to the best of our knowledge, nothing is known about whether such a bias of kicking direction can be observed in competitive soccer.

## Materials and Methods

### Participants

A total of 23 participants (nine females; mean age = 21.6 years; ranging from 18–27 years) with normal or corrected-to-normal vision took part in this experiment. All participants were sport science students at Bielefeld University and naïve to the purpose of the present experiment. However, none of the participants was an active soccer player, or had extensive practice in this sport. All reported to be right handed and right-footed. Before being tested, each participant gave his or her written informed consent. They were not paid for their participation. The study was approved by the local ethics committee and was carried out in accordance with the Helsinki Declaration of 1975.

### Apparatus and stimuli

A specific set-up was developed to take the penalty situation in soccer into the laboratory. To this end, the overall dimension of the set-up was downscaled to ∼44%, while keeping the relative distances constant to the real penalty situation. Accordingly, the goal was 3.19 m wide (real goal = 7.32 m) and 1.07 m high (real goal = 2.44 m). The penalty spot was placed at a distance of 4.80 m to the goal line (real penalty spot = 11.00 m). These dimensions were similar to the one reported by Masters et al., 2007, Experiments 2 and 3). All pictures were displayed onto a large, white wall with a projector, which was installed at a height of 2.60 m and a distance of 6.10 m to the projection wall. The position of the projector was carefully chosen, so that the view of the stimulus image was not obstructed by the participant during the task. At the same time, the stimuli were also visible on the experimenter’s laptop (shielded from view by the participant). In the action selection task, all shots were taken with a standard indoor soccer ball, made of hard foam.

**Figure 1:**
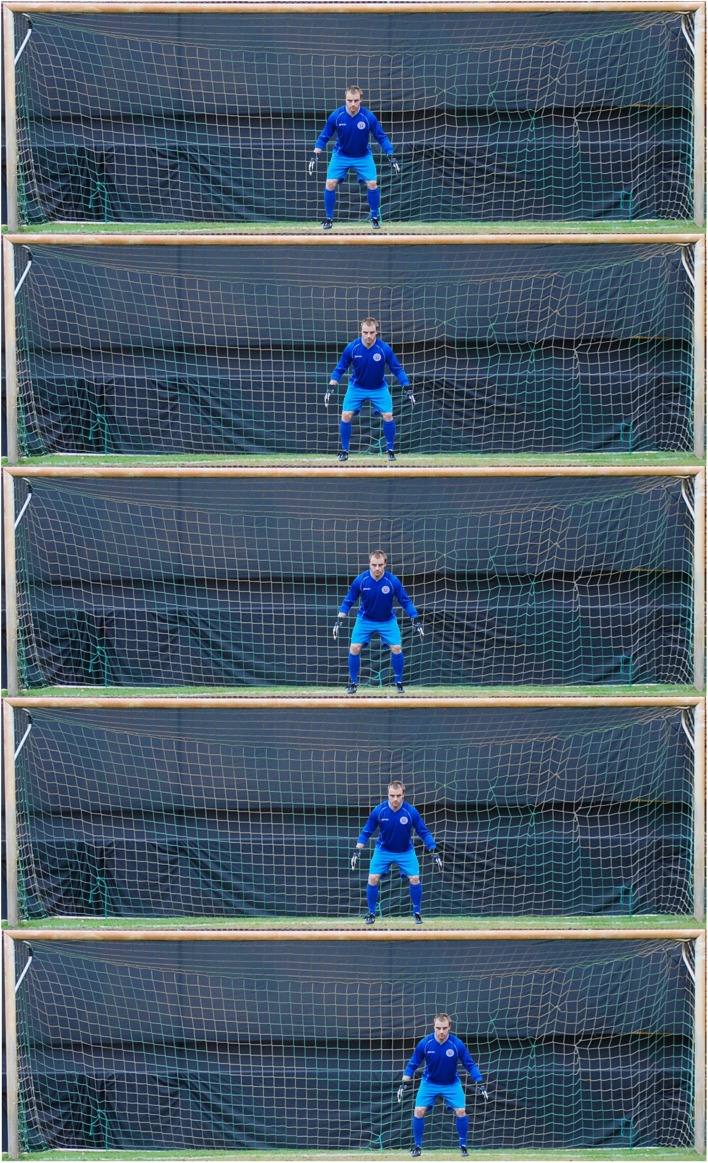
**Depicted are five of nine stimulus displays used in the present study**. The goal keeper was shown in a neutral goalkeeping posture, either in the goal’s center or in one of four displacements to the left (not displayed here) or right of center.

Stimuli were taken with a digital camera on an outdoor soccer pitch. They displayed a goalkeeper wearing a goalkeeper’s outfit and standing in a neutral goalkeeping posture in a standard size soccer goal. One picture was taken with the goalkeeper in the goal and one from the empty goal. Stimuli were then further edited on the PC with Corel Paint Shop Pro. To this end, the goalkeeper was carefully cut out and copied into the picture with the empty goal in one of nine positions, either in the goal’s center or 1.5, 3, 6, and 12% to the left and right of center (see Figure 1). This resulted in a total of nine stimulus images. The displacements of the goalkeeper to the left or right of the center relate to 11, 22, 44, and 88 cm in the realistic penalty situation.

### Experimental tasks

The experiment consisted of two parts in which participants performed an *action selection task* and a *perception task*. In both tasks, participants viewed a photo-realistic, static image of a goalkeeper, who stood either in the middle of the goal, or was marginally shifted to the left or right of the goal center. The goalkeeper was depicted in a neutral goal keeping posture (see Figure 1). The *action selection task* required participants to direct a penalty shot to the “open corner” of the goal (i.e., motor response). Similar to Masters et al., 2007, Experiment 2), they were instructed to select the side with the greater area uncovered. If they felt unsure about which side represented the greater area, they were instructed to follow their first impression and to take the shot without further contemplation. The *perception task* required participants to indicate (i.e., verbal answer) whether the goalkeeper stood in the middle, or was shifted to the left or right, respectively. Again, if they felt unsure about the actual position of the goalkeeper, they were instructed to follow their first impression.

### Design and procedure

All participants were tested first in the *action selection task*. They started with a practice block, in which each of the nine stimulus images was presented one at a time. The following test block consisted of 18 trials. Each trial started with the ball on the penalty spot and a blank screen for 1–2 s, whereupon, the experimenter presented the stimulus image on the screen. Then, the participant took a short approach of approximately 0.5–1.0 m and kicked the ball to the side, which she/he thought to present the greater area of the goal. Here it is important to note, that because participants were all right-footed, they approached the ball from the left side. After the shot was taken, the screen turned blank again and the ball was fetched (and returned to the penalty spot) by the participant. The next trial followed by presenting a new stimulus image. All stimulus images were presented in a pseudo-random order, which was predefined before the experiment and kept constant for each participant.

In the second part of the experiment, participants went on to be tested in the *perception task*. The procedure was similar to the first part, with one exception: The perception task did not require them to kick the ball to the goal, but instead to give a verbal judgment (i.e., explicit decision) of whether the goalkeeper stood in the middle of the goal, or to the left and right of the goal center. Thus, the instruction regarding the perception task was different to the instruction in the action selection task. The perception task was included in order to learn more about conscious and unconscious information processing and to derive a more explicit measure of participant’s perception of the goalkeeper’s position on the goal line. This aspect of the present study is different from the procedures of the original study by Masters et al. (2007), where participant’s perception of the goalkeeper’s position was inferred from individual confidence ratings (representing an indirect measure of participant’s perception). To this end, participants viewed each stimulus image while standing on the penalty spot. All stimulus images were presented in a new pseudo-random order.

Participants always started with the *action selection task* and then proceeded with the *perception task*. This order was not counterbalanced, because we wanted to avoid that participants would spend too much thought on the goalkeeper’s displacement in the first part of the experiment. In fact, the displacement of the goalkeeper was not mentioned to the participants before they actually started the perception task. Participants were allowed to take a short break between the two parts and the whole experiment lasted about 20 min.

### Data collection

Participant’s responses relative to the side of the goal (i.e., motor response) and the goalkeeper’s position (i.e., verbal response) were noted by the experimenter on two separate experimental score sheets, for the action selection task and the perception task, respectively. These score sheets contained the experimental schedule for the presentation of the stimulus images. Accordingly, the experimenter wrote down if the participant directed her/his kick to the left or right side of the goal in the first part of the experiment and where the participant perceived the goalkeeper standing in the second part. Thereby, kicking accuracy was of no further interest, so that all trials were also counted in which the ball would have actually missed the goal to the left or right.

### Data analyses

For the *action selection task*, data were analyzed for the different displacement conditions 1.5, 3, 6, and 12% of goal center. Since kicking side is a dichotomous variable, kicks to the smaller side of the goal were assigned a value of 0 and kicks to the greater side of 1. For each displacement condition, the sums of all trials were then divided by the number of trials times 100 to receive percentage values for kicking to the greater side for each participant. Planned comparisons (i.e., one-sample *t*-Tests) against chance level (50%) were conducted, beginning with the smallest displacement condition of 1.5% and continuing until a significant difference was reached. The data for the stimulus image in which the goalkeeper stood in the middle were analyzed separately to examine whether the (right-footed) participants had an implicit bias to direct their kicks to one or the other side of the goal.

For the *perception task*, participant’s judgments were analyzed to whether they indicated the goalkeeper to be in the middle of the goal, or not. Thus, the variable goalkeeper position was treated as a dichotomous variable, although participants could further indicate a displacement to the left or right side. Verbal judgments of left or right side of goal center received a value of 0, whereas a value of 1 was given when participants indicated the goalkeeper to be standing in the middle of the goal. The data for the stimulus image in which the goalkeeper was not displaced on the goal line (that is, zero-displacement) were analyzed separately to examine participants’ perceptual variability, e.g., perceiving the goalkeeper shifted to the left or right, when (in fact) he was not displaced, but stood in the exact middle of the goal. For the remaining conditions, the data was then further analyzed with planned comparisons (i.e., paired-samples *t*-Tests) between the zero-displacement condition and all displacement conditions, beginning with the smallest displacement condition of 1.5% and continuing until a significant difference was reached.

## Results

### Action selection task

When the goalkeeper was not displaced and presented in the exact middle of the goal, more kicks were directed to the right goal-side (27 out of 46 = 58.7% of the kicks) than to the left goal-side (19 out of 46 = 41.3% of the kicks). Accordingly, participants showed a kicking bias to the right goal-side. This kicking bias was in the magnitude of 17.4%.

**Figure 2:**
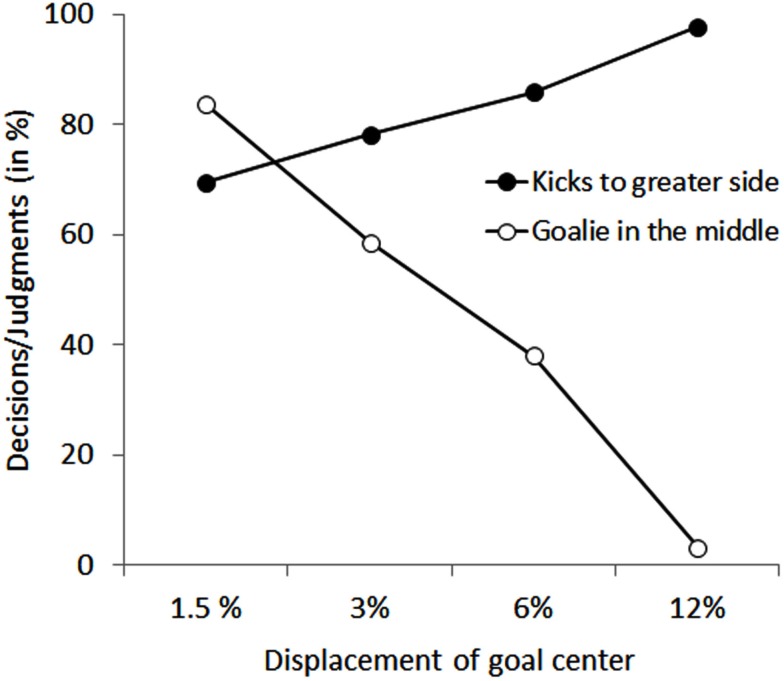
**Shows the pattern of results for kicks directed to the greater side of the goal as a function of goalkeeper’s displacement and participant’s expertise in the action selection task (AST, solid circles), as well as for judging the goalkeeper to be standing in the middle of the goal in the perception task (PT, open circles)**. Error bars indicate between-participant standard error.

Participant’s goal-side selections under conditions in which the goalkeeper was displaced along the goal line are shown in Figure 2. The solid circles represent the percentages of selecting the greater side of the goal for the different displacement conditions. The mean percentages for the different displacements were 69.6, 78.3, 85.9, 97.8%, from smallest to largest respectively. The planned comparison of the smallest displacement condition revealed a significant difference, *t*(22) = 3.600; *p *< 0.01. Hence, participants selected the larger goal-side already for the smallest displacement of the goalkeeper (i.e., 1.5% of goal center).

### Perception task

When the goalkeeper was not displaced and shown in the exact middle of the goal, participants indicated the middle position in the majority of trials (38 out of 46 trials = 82.6%), whereas on some occasions they reported the goalkeeper to be displaced (8 out of 46 = 17.4% of trials), irrespective of the fact that he was not moved.

Figure 2 shows the percentages of trials in which participants perceived the goalkeeper to be standing in the middle of the goal, although he was displaced along the goal line (open circles). As can be seen, the further the goalkeeper was standing off center, the less often participant’s perceived him in the middle. Accordingly, the percentages decreased from 83.7, 58.7, 38.0, to 3.3%. Planned comparisons between participants’ perceptual judgments in the zero-displacement condition and all displacement conditions revealed the following (none-)significant differences: For the 1.5% condition, participants perception was not different to the zero-displacement condition and thus, they were not aware of the displacement, *t*(22) = 0.182, *p *= 0.86. For the displacement condition of 3%, participant’s perceptual judgment of the goalkeeper position was significantly different from the zero-displacement condition. Hence, they became aware of the displacement when the goalkeeper was shifted by 3%, *t*(22) = 2.975, *p *< 0.01.

## Discussion

Anticipatory behavior does not only rely on well-established A-E representations, but also on the processing of the situational context in which these actions are carried out. When there is a high correspondence between the conditionalized A-E representations and the situational conditions, specific situational features will trigger the associated behavioral response by activating the conditionalized A-E representation. Hence, processing the action environment is essential for action selection. The general aim of the present study was to investigate whether an action instruction eases the selection of the open goal corner (when compared to a mere perception condition) as predicted by A-E theories (e.g., Hoffmann, 1993, 2009). Based on a recent study by Masters et al. (2007), who first reported implicit priming effects on goal-side selection, participants viewed photo-realistic images of a goalkeeper and a soccer goal. In the action selection task, they were asked to kick to the greater goal-side, whereas in the perception task, they indicated the position of the goalkeeper, who was either presented in the exact middle of the goal or was displaced at different distances to the left or right on the goal line. Two predictions were made: The general prediction related to implicit priming effects on participants’ goal-side selection and stated that participants would direct more kicks to the greater goal-side, even if they were not aware of the goalkeeper’s displacement. The specific prediction referred to a potential goal-side selection bias and predicted that the right-footed participants would kick more often to the right goal-side. The results of the present study are in line with these predictions and are discussed in the following.

With regard to the more general prediction of implicit priming effects on goal-side selection, the results of the action selection task confirmed that participants directed their kicks to the side of the goal with the greater area. This was already the case for the smallest displacement of 1.5% to the left or right, which corresponds to a distance of 11 cm in a real soccer goal. When asked to provide a verbal judgment of the goalkeeper’s position under this condition in the perception task, participants stated that they perceived the goalkeeper to be standing in the middle of the goal in the great majority of trials. Importantly, participants were not able to discriminate this small displacement of 1.5% from the zero-displacement condition (i.e., goalkeeper displayed in goal center). Therefore, it can be argued that they were not aware of the displacement. With larger displacements of the goalkeeper away from the goal’s center, participants more likely perceived him on the left or right side. They were becoming aware when the displacement was 3% and larger. This pattern of results replicates the implicit priming effects on penalty-kicking direction reported by Masters et al. (2007). It extends these findings, however, to a more realistic setting in which participants viewed photo-realistic images of a real soccer goal and a goalkeeper standing in a typical goalkeeping posture.

Another interesting observation was made for those stimuli, which displayed the goalkeeper to be standing in the exact middle of the goal. Here, participants indicated the goalkeeper to be standing in goal center in 82.6% of the trials. Participant’s performance was well above chance, but not perfect under this condition. At the same time, participants perceived the goalkeeper to be standing in the middle, when he was (in fact) displaced by a small degree (i.e., in 83.7% of trials under a 1.5% displacement). What may be the reason for this variability in the accuracy of participants’ perceptual judgments? It is possible that viewing natural scenes may have induced noise in the visual system and led to more (individual) variability in the processing of the spatial layout. Such individual variability during the visual processing of natural scenes has been reported elsewhere (Dorr et al., 2010) and may have affected participants’ judgment. Unfortunately, this pattern of results cannot be compared to the results of Masters et al. (2007), because these authors never presented the goalkeeper in the exact middle of the goal, even though participants were asked to kick only if they perceived the goalkeeper to be standing in goal center (Experiment 3). Thus, participants were instructed to respond to an experimental condition that was actually not included in the experiment. It can only be speculated that on some occasions, participants would not have responded, even if the authors had displayed the goalkeeper in the middle. This hypothetical result would be similar to the finding of perceptual variability in the present experiment.

With regard to the specific prediction referring to a potential goal-side selection bias, results showed that participants directed their kicks more often to the right side of the goal when the goalkeeper was presented in the exact middle. This goal-side selection bias may be explained by the biomechanics of kicking. Arguably, continuing to kick the ball in the direction of the run-up from left to right is easier in terms of skill execution (i.e., kicking the ball) than changing the kicking direction to the left goal-side. There is an alternative explanation for this goal-side selection bias, however. This alternative explanation is based on the observation that in about 17% of the trials in which the goalkeeper was presented in the middle of the goal, participants erroneously reported him to stand off center. If this perceptual “error” were systematic and included only mislocations to the left, then this may also explain the higher number of kicks to the right goal-side. However, when examining the participant’s judgments in the perception task for the smallest displacement (i.e., 1.5%) to the left side (goalie in the middle = 82.6%) and right side (goalie in the middle = 84.8%) separately and then comparing these number to the zero-displacement condition (goalie in the middle = 82.6%), a perceptual bias cannot be detected. Therefore, it seems rather unlikely, that the right-side kicking bias observed in the action selection task, being in the magnitude of 17.4%, can be explained by a perceptual bias. In any case, such a bias on goal-side selection has not been examined systematically for the penalty situation in competitive soccer. What has been reported, however, is that right-footed kickers score more often on the left goal-side (from their perspective), while left-footed kickers are more successful on the right side (Coloma, 2007). But this was not based on a more general kicking bias to one goal-side. More research is certainly needed to further determine the influence of the goal-side selection bias on kicking performance.

What kind of implications can be drawn for sports practice from the present experiment? For the penalty situation in soccer, specific performance-related instructions can be provided for goalkeepers to improve their success rate. Goalkeepers could use this knowledge about the implicit priming effect on goal-side selection strategically, by placing themselves a little more to their “weaker side” on the goal line. This will increase the likelihood of the penalty taker to kick to the opposite side of the goal, which corresponds with the “stronger side” of the goalkeeper. A goalkeeper displacement of 1.5% resulted in roughly 69.6% of kicks to the greater goal-side. At the same time, this small displacement, which corresponds to 11 cm in the real-size soccer goal, was not perceived by the participants, neither in the present study nor in the study of Masters et al. (2007). Instead of such a strategic displacement on the goal line, goalkeepers can also use explicit gestures. For example, an active goalkeeper that moves and waves her/his arms around can effectively distract the penalty taker (Wood and Wilson, 2010). Also, explicit signaling with specific pointing gestures to the left or right goal-side can render the upcoming kicking direction more predictable (Weigelt et al., in press).

In summary, decision making in soccer penalty kicking (i.e., goal-side selection) can be systematically influenced by the goalkeeper through the utilization of implicit (i.e., goalkeeper displacement) information strategies. The present experiment therefore adds further empirical evidence to the growing body of research on perception-action-coupling, using the soccer penalty situation as an experimental paradigm (e.g., Masters et al., 2007; Dicks et al., 2010a,b). It extends previous research by using photo-realistic stimulus material. The present findings provide valuable implications for specific performance-related instructions to benefit the performance of goalkeepers and penalty takers. To take the present findings from the laboratory to the field, future studies should examine the effectiveness of such performance-related instructions with representative task designs (cf. Dicks et al., 2009).

## Conflict of Interest Statement

The authors declare that the research was conducted in the absence of any commercial or financial relationships that could be construed as a potential conflict of interest.
